# Comparing critical speed modelling approaches and exploring relationships with match-play variables in elite male youth soccer players

**DOI:** 10.17159/2078-516X/2021/v33i1a9738

**Published:** 2021-07-01

**Authors:** KC Liu, J Sheard, T Frixou, P Milton, E Prato Luna, E Piatrikova, S Williams, J Parr, G Roe, M Kramer

**Affiliations:** 1 Department of Health, University of Bath, Bath, UK; 2 Manchester United Football Club, Manchester, UK; 3 Bath Rugby, Bath, UK; 4 Physical Activity, Sport, and Recreation (PhASRec) Unit, North-West University, Potchefstroom, South Africa

**Keywords:** bi-exponential, critical power, reference, performance

## Abstract

**Background:**

A novel bi-exponential method has emerged to estimate critical speed (CS) and D-prime (*D*′) from a 3-min all-out test (3MT).

**Objectives:**

To compare CS analysis methods to determine whether parameter estimations were interchangeable. Reference values and relationships with key soccer match-play variables were explored.

**Methods:**

Thirteen elite male youth (14–15 years old) players completed a 30 m shuttle run 3MT to estimate CS, *D*′, rate of speed decline time constant, maximal speed (*S*_max_), time to *S*_max_ (*t*_max_), and fatigue index (FI), using the traditional method and bi-exponential model on average (Bi-Exp_Average_) and max speed settings (Bi-Exp_Max-Speed_). High-speed running (HSR) and sprinting distances and counts, and the number of accelerations were collected from two matches. Magnitude-based inferences (*p* < 0.05) with smallest worthwhile change of 0.2 effect sizes were used to analyse differences. Pearson’s and Spearman’s correlation coefficients were used to measure associations between CS model variables and match-play parameters.

**Results:**

There were significant differences between the traditional method and both bi-exponential models for CS and *D*′, as well as between the bi-exponential models for all variables except *t*_max_. Using the Bi-Exp_Average_ model, strong correlations (*r* = 0.70–0.73; *p* < 0.05) were observed for *D*′ and FI with the number of standardised and individualised HSRs, respectively. With the Bi-Exp_Max-Speed_ model, there were strong correlations (*r*/ρ = 0.64–0.68; *p* < 0.05) between *D*′ and the number of standardised HSRs and sprints, and the number of individualised sprints.

**Conclusion:**

There is a lack of interchangeability between analysis methods. It appears that *D*′ and FI from the bi-exponential models could be associated with high-intensity actions in soccer match-play.

First interpreted by Hill in 1925, the concept of critical power (CP) is mathematically described as the hyperbolic relationship between an individual’s sustainable power output during exercise and time to exhaustion.^[1]^ CP is defined as the horizontal asymptote of power output on a power-time curve, below which exercise can be sustained for an extended (theoretically unlimited; in practice 20–60 mins) period of time.^[2]^ Exercise at or above CP; however, draws upon the individual’s additionally available work capacity represented by *W*′ (expressed in kJ), the magnitude of which is finite and its depletion predictable.^[2]^ In sports, such as cycling and rowing, three-point models from fixed-distance time trials are traditionally used to map an athlete’s power-time relationship.^[3]^ However, conducting multiple tests is time-consuming, labour-intensive, and aversive for the participants. This has been addressed through the development of a three minute all-out test (3MT) that provides valid and reliable estimates of CP and *W′* as a single maximal test for both power and speed-based sports.^[2,4]^ The average power output in the last 30 seconds of the 3MT was shown to decline to a relatively steady level that was almost identical to CP.^[5]^ Based on the linearisations of the power-time relationship,^[6]^ the derived equation is expressed as:


$$W^{\prime }=150s({\text{P}}_{150\text{s}}-\text{CP})$$

where P_150s_ is the average power output across the first 150 seconds. This relationship also exists in running, where the terms critical speed (CS) and D-prime (*D*′) are used instead.^[2]^ Research on collegiate female distance runners has also shown that the 3MT is equally effective for measurements of CS and *D′*,^[5]^ but there was a lack of interchangeability of results from a linear 3MT to an intermittent repeated-sprint based scenario. Although the average running speed for repeated sprints is lower, there is a higher energy cost of accelerations from intermittent running,^[7]^ where oxygen consumption (V^·^O_2_) requirements increase in proportion to speed and turn frequency.^[8]^ Nevertheless, recent evidence has shown that the 3MT can be modified to a shuttle run protocol that is equally valid to continuous running models. A length of 30 m allowed enough time to build up to near-maximal speeds, but was short enough to allow for a considerable number of turns.^[9]^

In a recent study on team sport athletes, a novel bi-exponential model has emerged as another method of quantifying elements of the running 3MT, showing very strong and fit aspects to the data (*r* = 0.91–0.97) and high levels of agreement for estimates of CS and *D*′ against a graded exercise test.^[10]^ For the shuttle-run 3MT, there are two methods of bi-exponential modelling that can be used to estimate the measures: an average speed method (Bi-Exp_Average_) that calculates CS using average speed per shuttle; and a maximum speed method (Bi-Exp_Max-Speed_) that uses peak shuttle speeds only. The bi-exponential model also reported additional physiological parameters of interest that can be obtained from the data (Fig. 1), including *S*_max_ (maximum speed of the trial), *t*_max_ (time to *S*_max_), τ_d_ (time constant reflecting rate of speed decline towards CS), and *A*_d_ (amplitude of decline from *S*_max_ to CS). Since *A*_d_ effectively describes a reserve for speeds above CS, it was used to derive a fatigue index (FI), defined as the speed reserve as a percentage of *S*_max_.^[10]^ It was suggested that smaller FI values represented lower levels of fatigability, thus a disposition to endurance exercise.^[10]^

Due to the novelty of the bi-exponential model, comparisons with existing shuttle running evidence have not yet been established, and so the interchangeability of results between methods is currently unclear. Although research on CS has mainly been applied to soccer^[6,11]^ and rugby,^[4,8]^ to date only one study has explored the use of the CS concept to the classification of match running performance.^[12]^ To our knowledge, there are currently no studies that have examined the relationship between CS parameters (as derived from the 3MT) and key match-play physical performance variables in team sports. Therefore, the three-fold objective of this study was to provide specific insights based on a soccer population. The first objective was to compare CS parameters obtained using different models from the shuttle running 3MT, when compared between the traditional, Bi-Exp_Average_, and Bi-Exp_Max-Speed_ analysis methods. The second objective was to establish reference values from all three analysis methods. The final objective was exploratory, namely to provide novel insight into possible relationships between CS parameters and key match-play variables.

## Methods

### Participants

Thirteen competitive, elite youth male soccer players (age 15.2±0.2 years, height 171.3±7.0 cm, weight 59.0±7.8 kg) from an English Premier League academy were recruited. Pacing was detected in the 3MT time trial from one player and so his data were excluded, resulting in a total of twelve players. Testing occurred around the middle of the season, and all participants were engaged in training, as well as strength and conditioning programmes, at the time. As all athletes were under the age of 16 years old, written informed consent was given by their parents or guardians along with written informed assent from the athletes, and approved by the University of Bath Research Ethics Approval Committee for Health in accordance with the Declaration of Helsinki.

### Protocol

The current study is of a cross-sectional observational nature. The participants underwent two shuttle-run 3MT trials in the evening under dry weather conditions on a grass surface. The trials were separated by 72 hours to allow for sufficient recovery, and the participants did not take part in any matches for at least 48 hours prior to each trial. The first trial was used as a familiarisation part of the trial, with the second acting as the main trial for data collection. The protocols consisted of continuous maximal effort, namely, 30 m shuttle sprints for a duration of 185 seconds, without any indication of time remaining throughout to prevent pacing. The trials were conducted at the beginning of training after a regular warm-up to prevent fatigue from influencing the results. An extra five seconds were added to ensure that the three minutes of data were available for all participants. Participants also completed two 80-minute matches with a 15-minute break between halves, 48–60 hours prior to each trial.

Data from the trials and matches were collected using an 18 Hz global positioning system’s (GPS) units (Apex Pro Series, STATSports, County Down, Northern Ireland), positioned between the participants’ scapulae in a tight-fitted vest. The accompanying software was used for data extraction. For the 3MTs, instantaneous speed (m·s^−1^) was obtained for every time point. The data were processed using the traditional method and the bi-exponential models in an Excel spreadsheet^[10]^ to calculate CS, *D*′, *S*_max_, *t*_max_, τ_d_, and *A*_d_, based on methods presented in other published literature.^[5,10]^

Match-play variables were obtained for the final 15 minutes of each half only, as the disparity between physical capabilities is more likely to be differentiated when fatigue starts to influence output.^[13]^ The metrics obtained were high-speed running (HSR; ≥5.5 m·s^−1^) and sprinting (≥7.0 m·s^−1^) distances, and number of accelerations (≥2.0 m·s^−2^). A Python script (Connor, 2020) was also employed to obtain the number of HSR and sprinting counts based on the standardised thresholds, as well as HSR and sprinting distances and counts based on individualised thresholds. Each player’s CS and *S*_max_ were used as their individualised HSR and sprinting thresholds respectively, which were repeated for all three analysis methods. Dwell time was set at 0.5 seconds for an action count to register. Each half of the matches was treated separately, and only data from players who started the half were included to ensure playing time leading up to the last 15 minutes was equal. Data from five players who participated in both match fixtures were averaged prior to inclusion in the final analysis.

### Statistical analysis

Normality of all data were assessed with the Shapiro-Wilk test. To answer the first objective, we utilised magnitude-based inferences (MBI) with a 95% confidence level, alpha-level of *p* = 0.05, and smallest worthwhile change of 0.2 for Cohen’s *d* effect sizes (ES) to evaluate the differences between variables estimated from the traditional and bi-exponential methods. ES values were interpreted using the following scale: d < 0.2, trivial; < 0.6, small; < 1.2, moderate; < 2.0, large; < 4.0, very large; and ≥ 4.0, extremely large.^[14]^ For the second objective, reference values were calculated using averages (i.e. mean ± standard deviation) for all three methods.

Finally, in alignment with the third objective, we used Pearson’s or Spearman’s correlation coefficients to explore relationships between physiological variables from the 3MT shuttle-run test and match-play metrics. The correlation test was chosen based on the normality of each dataset. Specifically, CS was analysed against distance measures, *D*′ against action counts, and FI against all metrics. Correlation values were interpreted using the following scale: r/ρ < 0.2, very weak; < 0.4, weak; < 0.6, moderate; < 0.8, strong, and ≥ 0.8, very strong.

## Results

### Comparison between methods of analysis

The differences in chosen CS variables between methods of analysis are reported with Cohen’s *d* and derived MBIs in Tables 1, 2, and 3, along with raw values and ES interpretations. There were statistically significant differences in all variables between the traditional method and both bi-exponential models. Comparisons between the two bi-exponential models also found statistically significant differences in all variables except for *t*_max_.

### Reference values

Reference values are represented by squad averages in Table 4, along with standard deviations and 95% confidence intervals. Results from all three methods of calculations are shown separately.

### Relationships between key variables

Exploratory correlations between CS, *D*′, FI, and key match-play variables from the last 15-minute periods of each half are shown in Tables 5, 6, and 7. No statistically significant correlations were found between CS and distances covered above high-intensity running-speed thresholds. *D*′ derived from both bi-exponential models showed strong correlations to the number of standardised HSR efforts. Similarly, strong relationships were found between *D*′ from the Bi-Exp_Max-Speed_ model and both the number of standardised and individualised sprinting efforts. Finally, FI from the Bi-Exp_Average_ model also exhibited a strong correlation to the number of individualised HSR actions.

## Discussion

The key results of this study are threefold. Firstly, comparisons between the three calculation methods show that there are small to extremely large differences in all values, with the exception of *t*_max_ being possibly trivial. Next, reference values were presented for all variables for a male elite youth soccer population. Finally, strong correlations were found between bi-exponential *D*′, FI, and certain measures of HSR efforts.

The differences in CS values between the bi-exponential and traditional methods can be attributed to the fact that the bi-exponential models detect when shuttle speeds level off, instead of calculating from a standardised final 30 seconds. Indeed, the 0.71 m·s^−1^ larger CS from the Bi-Exp_Max-Speed_ model versus the traditional method can be explained by the model’s use of only shuttle peak speeds, thus excluding the periods of lower velocity during accelerations and decelerations.

For *D*′ from the Bi-Exp_Average_ and Bi-Exp_Max-Speed_ models, the 67.84 m larger and 13.58 m smaller mean, respectively, can be explained by the bi-exponential models calculating the area under the curve rather than averaging the speed from the initial 150 seconds. Due to the larger CS from the Bi-Exp_Max-Speed_ model, the lower *D*′ values are in line with the well-established inverse relationship between the two parameters.^[2,3]^ As CS is higher for the same individual maximal oxygen uptake (V^·^O_2max_), the range of available work capacity above the threshold is accordingly reduced. The larger *S*_max_ and FI averages from the Bi-Exp_Max-Speed_ compared with the Bi-Exp_Average_ model were also to be expected, due to the nature of the speed values and the calculation formulas used. This is supported by a study that found larger speed decrements when max speed is higher^[15]^ suggesting a lower level of endurance that can be represented by the larger FI values seen.

Although parameter differences between models may appear to be purely semantic, it is important to remember that CS and D′ have been successfully used in individualised training prescription.^[5]^ Therefore, the magnitude of CS and D′ derived from a model can have significant influences in time-to-exhaustion efforts, as well as performance and training success at the individual level. The present study therefore highlights the meaningful differences in parameter estimates, and future research should therefore investigate which model provides more useful shuttle-based CS and D′ parameters for training prescription.

Together, the present data implies that results obtained from different calculation methods cannot be interpreted interchangeably, especially for measures of CS, *D*′, *S*_max_, and FI. Sports science practitioners and researchers should only make comparisons for data derived from the same calculation method, otherwise conclusions will be erroneous and inaccurately reflect the nature of any physiological differences within individuals and between groups. Based on the importance of accelerations and decelerations to physical performance in soccer^[16]^, the use of the traditional method or Bi-Exp_Average_ model may be more appropriate for training prescriptions to be able to mimic match-play demands. On the other hand, the Bi-Exp_Max-Speed_ model could serve better for longitudinal monitoring purposes, as its focus on shuttle peaks may allow for the capture of loads above speed thresholds with larger physiological impacts. As it had been shown that shuttle-run 3MTs of varying distances produces differences in measures,^[8,11]^ future research should investigate comparisons using different shuttle lengths that are more suitable to other team sports to determine whether similar discrepancies between the analysis methods exist.

Reference data were presented in this study for an elite male U16 soccer population. Aside from the well-established CS and *D*′ metrics, additional physiological parameters introduced by the bi-exponential models were also reported. As τ_d_ is the time constant reflecting the rate of speed decline, higher values can possibly represent a better speed endurance ability in maintaining speeds above CS. Lower values of *t*_max_ can suggest a greater accelerating capability to reach top speed within 30 m, represented by a higher *S*_max_. Finally, FI can be used as a measure of fatigability, with a lower value suggesting a propensity for higher endurance capacity.^[10]^ These qualities are critical to performance in not only soccer, but also across a variety of intermittent sports.

A recent quasi meta-analysis on field sports athletes based on the traditional method found a mean CS of around 3.5 m·s^−1^ and *D*′ of around 225 m.^[17]^ Although CS is comparable to the mean of 3.42 m·s^−1^ in this study, *D*′ was much larger than the 58.82 m from our population. However, a key point to consider is that the average participant age from the four studies included ranged from 19–24 years. The difference in maturation status in comparison to U16 players might explain at least some of the discrepancies. Three of the studies used varying shuttle distances ranging from 20–50 m, and one in fact conducted a linear 3MT instead, whilst two included female participants. As a result, comparisons drawn between studies cannot be conclusive, as research has shown that measure outcomes are specific to 3MT protocols,^[8]^ and gender differences were not addressed.

Lastly, the exploratory correlations examined provide an insight into possible relationships between measures of the CS concept and key match-play variables. Several statistically significant associations, which could be considered strong based on the correlation coefficients, were found between bi-exponential *D*′, FI, and measures of HSR and sprint counts. Since *D*′ and FI represent work capacity and fatigability respectively, positive associations with high-intensity actions in latter stages of each half can be deemed reasonable. These ideas are in line with research that found negative effects of fatigue on high-intensity activity in the latter stages of halves,^[13]^ which can in turn suggest that larger physiological capacities allow for more pronounced physical performance capabilities. No significant relationships were found with the other variables explored. Neither bi-exponential models seemed to show stronger links than the other. The small number of effects observed can be attributed to several limitations within this study, mainly the limited sample size of participants. There was also a lack of consideration for match status during the 15-minute periods, as being in a winning, drawing, or losing position can affect motivation to engage in high-intensity activity.^[18]^ The team’s tactical approaches and prior success,^[19]^ quality of opposition, as well as each individual’s status (e.g. recovery and wellness) may also affect physical output. It is hoped that the results of the present study may provide impetus for future research pertaining to the relationship between parameters derived from the 3MT and actual match-play as such information is currently lacking.

## Conclusion

In conclusion, the analysis of shuttle-run 3MTs using the traditional method and bi-exponential models produced significantly different values for all but one metric calculated. Therefore, results are not interchangeable and researchers must interpret data from existing literature with some caution. The male youth soccer reference values provided may guide practitioners in fitness diagnostics, performance evaluation, and training prescriptions. Exploratory correlations found between key variables suggest possible relationships exist between bi-exponential *D*′, FI, and high-intensity running actions in match-play. This proposes applicability to the appraisal of a player’s ability to perform actions that can affect match outcomes. Finally, results and ideas presented in this study warrant more extensive research, and scope for future investigations have been discussed.

## Figures and Tables

**Fig. 1:**
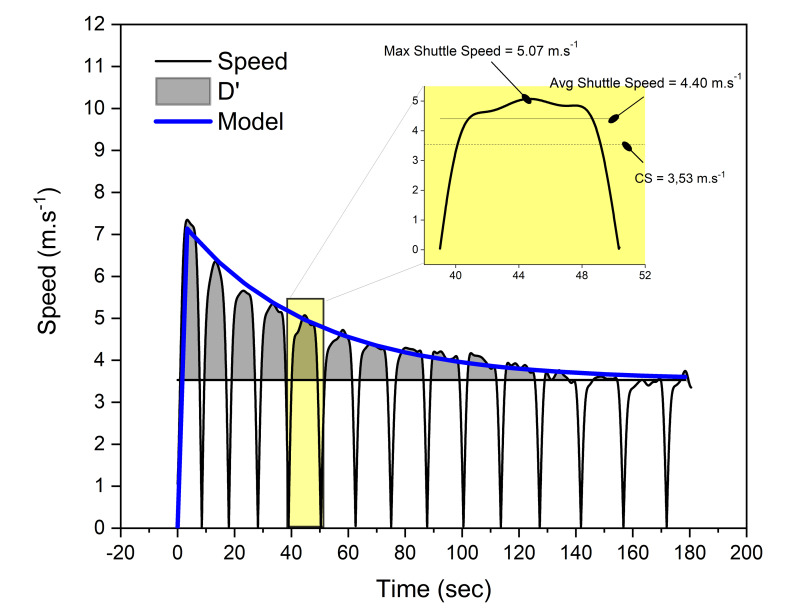
Example of a speed-time graph of a running 3-minute all-out test analysed using the bi-exponential model. The highlighted shuttle demonstrates the difference between max and average (avg) speeds used for modelling. CS, critical speed; D′, D-prime

**Table 1:** Comparisons of variables between traditional method and Bi-Exp_Average_ model

Variable	Mean*_diff_*	*p*-value	Cohen’s *d*	95% CI	Effect size interpretation	MBI
CS (m·s^−1^)	−0.07	0.02	−0.42	−0.76, −0.08	Small	Likely negative
*D*′ (m)	67.84	< 0.01	4.16	3.41, 4.91	Almost certain	Most likely positive

Bi-Exp_Average_, average speed bi-exponential; Mean_diff_, mean of Bi-Exp_Average_ model – mean of traditional method; CS, critical speed; D′, D-prime; CI, confidence intervals; MBI, magnitude-based inferences. Negative MBI denotes a larger value in the traditional method.

**Table 2:** Comparisons of variables between traditional method and Bi-Exp_Max-Speed_ model

Variable	Mean*_diff_*	*p*-value	Cohen’s *d*	95% CI	Effect size interpretation	MBI
CS (m·s^−1^)	0.71	< 0.01	3.85	3.46, 4.25	Very large	Most likely positive
*D*′ (m)	−13.58	0.03	−0.86	−1.61, −0.11	Moderate	Very likely negative

Bi-Exp_Max-Speed_, max speed bi-exponential; Mean_diff_, mean of Bi-Exp_Max-Speed_ model – mean of traditional method.; CS, critical speed; D′, D-prime; CI, confidence intervals; MBI, magnitude-based inferences. Negative MBI denotes a larger value in the traditional method.

**Table 3:** Comparisons of variables between Bi-Exp_Average_ and Bi-Exp_Max-Speed_ models

Variable	Mean*_diff_*	*p*-value	Cohen’s *d*	95% CI	Effect size interpretation	MBI
CS (m·s^−1^)	0.78	< 0.01	4.41	4.10 to 4.71	Extremely large	Most likely positive
*D*′ (m)	−81.41	< 0.01	−7.06	−7.41 to −6.71	Extremely large	Most likely negative
τ_d_ (s)	−3.88	0.09	−0.39	−0.86 to 0.07	Small	Likely negative
*t*_max_ (s)	−0.24	0.42	−0.12	−0.45 to 0.20	Trivial	Possibly trivial
*S*_max_ (m·s^−1^)	1.74	< 0.01	6.87	6.20 to 7.55	Extremely large	Most likely positive
FI (%)	6.8	< 0.01	1.41	1.09 to 1.74	Large	Most likely positive

Bi-Exp_Average_, average speed bi-exponential; Bi-Exp_Max-Speed_, max speed bi-exponential; Mean_diff_, mean of Bi-Exp_Max-Speed_ – Bi-Exp_Average_ model.; CS, critical speed; D′, D-prime; τ_d_, time constant reflecting rate of speed decline towards CS; t_max_, time to S_max_; S_max_, maximum speed; FI, fatigue index; CI, confidence intervals; MBI, magnitude-based inferences. Negative MBI denotes a larger value in the traditional method.

**Table 4:** Reference values of traditional method, Bi-Exp_Average_, and Bi-Exp_Max-Speed_ models

Variable	Traditional method			Bi-Exp_Average_ model			Bi-Exp_Max-Speed_ model		

	Mean	SD	95% CI	Mean	SD	95% CI	Mean	SD	95% CI
CS (m·s^−1^)	3.42	0.17	3.31 to 3.51	3.35	0.15	3.27 to 3.44	4.13	0.20	4.01 to 4.24
*D*′ (m)	58.82	19.52	47.77 to 69.86	126.65	12.29	119.70 to 133.61	45.24	10.71	39.18 to 51.30
τ_d_ (s)	-	-	-	37.99	9.97	32.35 to 43.63	34.11	9.69	28.63 to 39.59
*t*_max_ (s)	-	-	-	4.70	2.00	3.57 to 5.83	4.46	1.86	3.41 to 5.51
*S*_max_ (m·s^−1^)	-	-	-	5.00	0.11	4.94 to 5.06	6.74	0.34	6.54 to 6.93
FI (%)	-	-	-	39.0	5.1	36.1 to 41.9	45.8	4.6	43.2 to 48.4

Bi-Exp_Average_, average speed bi-exponential; Bi-Exp_Max-Speed_, max speed bi-exponential; CS, critical speed; D′, D-prime; τ_d_, time constant reflecting rate of speed decline towards CS; t_max_, time to S_max_; S_max_, maximum speed; FI, fatigue index; CI, confidence intervals; MBI, magnitude-based inferences. Negative MBI denotes a larger value in the traditional method.

**Table 5:** Correlation coefficients between critical speed and match-play distance measures

Method	Std. HSR	Ind. HSR	Std. Sprint	Ind. Sprint
Traditional	0.37	0.04	0.05	-
Bi-Exp_Average_	0.25	0.11	0.04	0.17
Bi-Exp_Max-Speed_	0.32	−0.19	0.10	0.15

Bi-Exp_Average_, average speed bi-exponential; Bi-Exp_Max-Speed_, max speed bi-exponential; Ind, individualised; Std. HSR, standardised high-speed running (≥ 5.5 m·s^−1^); Std. Sprint, standardised sprint (≥ 7.0 m·s^−1^);

* p < 0.05.

**Table 6:** Correlation coefficients between *D*′ and match-play action count measures

Method	No. of Std. HSR	No. of Ind. HSR	No. of Std. Sprint	No. of Ind. Sprint
Traditional	−0.19	0.14	0.14	-
Bi-Exp_Average_	0.73*	0.52	0.36	−0.33
Bi-Exp_Max-Speed_	0.68*	0.34	0.64*	0.64*

Bi-Exp_Average_, average speed bi-exponential; Bi-Exp_Max-Speed_, max speed bi-exponential; Ind, individualised; Std. HSR, standardised high-speed running (≥ 5.5 m·s^−1^); Std. Sprint, standardised sprint (≥ 7.0 m·s^−1^);

* p < 0.05.

**Table 7:** Correlation coefficients between fatigue index (FI) and match-play key variables of interest

Method	Std. HSR	Ind. HSR	Std. Sprint	Ind. Sprint	No. of Std. HSR	No. of Ind. HSR	No. of Std. Sprint	No. of Ind. Sprint	No. of accelerations
Bi-Exp_Average_	0.12	0.02	0.28	0.06	0.56	0.70*	0.50	−0.24	< 0.01
Bi-Exp_Max-Speed_	0.29	0.11	0.52	−0.11	0.52	0.59	0.57	0.57	−0.09

Bi-Exp_Average_, average speed bi-exponential; Bi-Exp_Max-Speed_, max speed bi-exponential; Ind, individualised; Std. HSR, standardised high-speed running (≥ 5.5 m·s^−1^); Std. Sprint, standardised sprint (≥ 7.0 m·s^−1^);

* p < 0.05.
